# Impact of remote monitoring in heart failure patients with cardiac implantable electronic devices during COVID-19 pandemic: a single center experience

**DOI:** 10.1186/s13019-022-01963-y

**Published:** 2022-08-28

**Authors:** Péter Ezer, Marin Gergics, István Szokodi, Attila Kónyi

**Affiliations:** 1 Heart Institute of the Clinical Center University of Pécs, University of Pécs Medical School, Ifjuság Street 13, Pecs, 7621 Hungary; 2 1st Department of Medicine of Clinical Center University of Pécs, University of Pécs Medical School, Ifjusag ut 13, 7621 Pecs, Hungary; 3 Szentágothai Research Center, University of Pécs, Ifjusag ut 13, Pecs, 7621 Hungary

**Keywords:** Heart failure, Remote monitoring, Follow-up, COVID-19

## Abstract

**Background:**

Coronavirus disease 2019 (COVID-19) had spread into a pandemic affecting healthcare providers worldwide. Heart failure patients with implanted cardiac devices require close follow-up in-spite of pandemic related healthcare restrictions.

**Methods:**

Patients were retrospectively registered and clinical outcomes were compared of 61 remote monitored (RMG) versus 71 conventionally (in-office only) followed (CFG) cardiac device implanted, heart failure patients. Follow-up length was 12 months, during the COVID-19 pandemic related intermittent insitutional restrictions. We used a specified heart failure detection algorithm in RMG. This investigation compared worsening heart failure-, arrhythmia- and device related adverse events as primary outcome and heart failure hospitalization rates as secondary outcome in the two patient groups.

**Results:**

No significant difference was observed in the primary composite end-point during the first 12 months of COVID-19 pandemic (*p* = 0.672).

In RMG, patients who had worsening heart failure event had relative modest deterioration in heart failure functional class (*p* = 0.026), relative lower elevation of N terminal-pro BNP levels (*p* < 0.01) at in-office evaluation and were less hospitalized for worsening heart failure in the first 6 months of pandemic (*p* = 0.012) compared to CFG patients.

**Conclusions:**

Specified remote monitoring alert-based detection algorithm and workflow in device implanted heart failure patients may potentially indicate early worsening in heart failure status. Preemptive adequate intervention may prevent further progression of deteriorating heart failure and thus prevent heart failure hospitalizations.

## Background

The outbreak of the coronavirus disease 2019 (COVID-19) had spread into a pandemic situation affecting healthcare providers around the world. In the spring of 2020 healthcare systems were warned to potentially decrease the number of institutional in-office patient evaluations (IPE) to reduce human contacts and thus potential further spread of COVID-19. In this manner the pandemic related healthcare restrictions had limited the patients physical contact to the medical staff.

COVID-19 fundamentally altered healthcare logistics and patient access to healthcare services. Furthermore even healthcare workers were prone to persistently increasing viral transmission rate affecting up to 29% of all active workers in this field in Italy [1].

Remote monitoring (RM) has revolutionized the follow-up of cardiac implantable electronic device (CIED) patients in the last 20 years. Prespecified device alerts—depending on the manufacturer of the system—provide support to follow certain physiological parameters, alert device malfunction, arrhythmia events and even deterioration in the patients heart failure status reliably. This mode of detection promotes rapid response for urgent clinical and device technical issues thus leading to improved patient outcomes [2, 3]. Some studies of automated daily remote monitored advanced heart failure CIED patients resulted even in improved survival compared to conventional – IPE based – care [4, 5].

Detecting worsening heart failure remains one of the main trending issues in remote patient monitoring, although previously an upgraded remote patient monitoring based heart failure detection algorithm was published by Whellan et al. in PARTNERS HF trial [6] and was optimalized by Vámos et al. [7]. This alert based follow-up algorithm seems accurate enough to predict an upcoming heart failure event with sensitivity about 86.5% and specificity of 93%.

Expert recommendations emphasized the potential benefits of remote monitoring in non-CIED heart failure patient group for potential better and safer patient management during COVID-19 pandemic related healthcare restrictions [8, 9] and expert position statements were published for reducing in-office patient evaluation follow-up burden and face-to-face visit events resulting in potential minimised exposure of patients and healthcare workers [8, 10–12]. Some authors suggested consequent activation of RM function in all newly implated CIEDs [13], or declared RM as essential in the follow-up of CIED patients during the pandemic [14, 15]. Aim of this study was to investigate, wheter symptomatic heart failure patients, with implanted defibrillators (ICD) or cardiac resynchronization therapy pacemakers (CRT-P) or defibrillators (CRT-D) capable to remote follow-up may have clinical benefits in terms of rapid detection of worsening heart failure or other clinival adverse events compared to a conventionally followed (non-monitored) patient group during the special scenario of COVID-19 pandemic.

## Methods

Data were retrospectively acquisited of 132 patients implanted with single- or dual chamber ICD, CRT-D or CRT-P devices. All the patients involved in this study were implanted for at least 1 year before March of 2020 and were in NYHA II or III functional class at the beginning of the follow-up period. Device implantations were all performed in consenus with currently available guidelines of European Society of Cardiology for device therapy and heart failure [16]. Remote monitoring group (RMG) consisted of 61 patients whereas conventionally followed group (CFG) consisted of 71 patients. Follow-up period was 12 months from 15.03.2020 until 15.03.2021. Data collection was performed in accordance with international regulations regarding the protection of personal information and data. All subjects gave their informed consent for inclusion before they participated in the study and agreed of anonymous scientific use of their data. The study was conducted in accordance with the Declaration of Helsinki and meets the ethical standards and is in accordance with the guidelines provided by the CPCSEA and World Medical Association Declaration of Helsinki on Ethical Principles for Medical Research Involving Humans. The studyprotocol was approved by the Ethics Committee of the University of Pécs, Hungary (Ethical serial number: 6600/2020).

Patients in the RMG had *Biotronik Home Monitoring*™ or *Medtronic Care Link*™ RPM eligible devices. CFG patients have been implanted with devices from various manufacturers: *Biotronik*™, *Medtronic*™, *Boston Scientific*™, and *St Jude Medical*™ without the capability for RPM function.

### Prespecified remote patient monitoring algorithm for worsening heart failure

Effective remote patient management (RPM) via CIEDs has long been achievable but due to lack of adoption of easy manageable algorithm-driven alert-based systems and absence of randomized protocols this technology was underutilized until now.

**Fig. 1:**
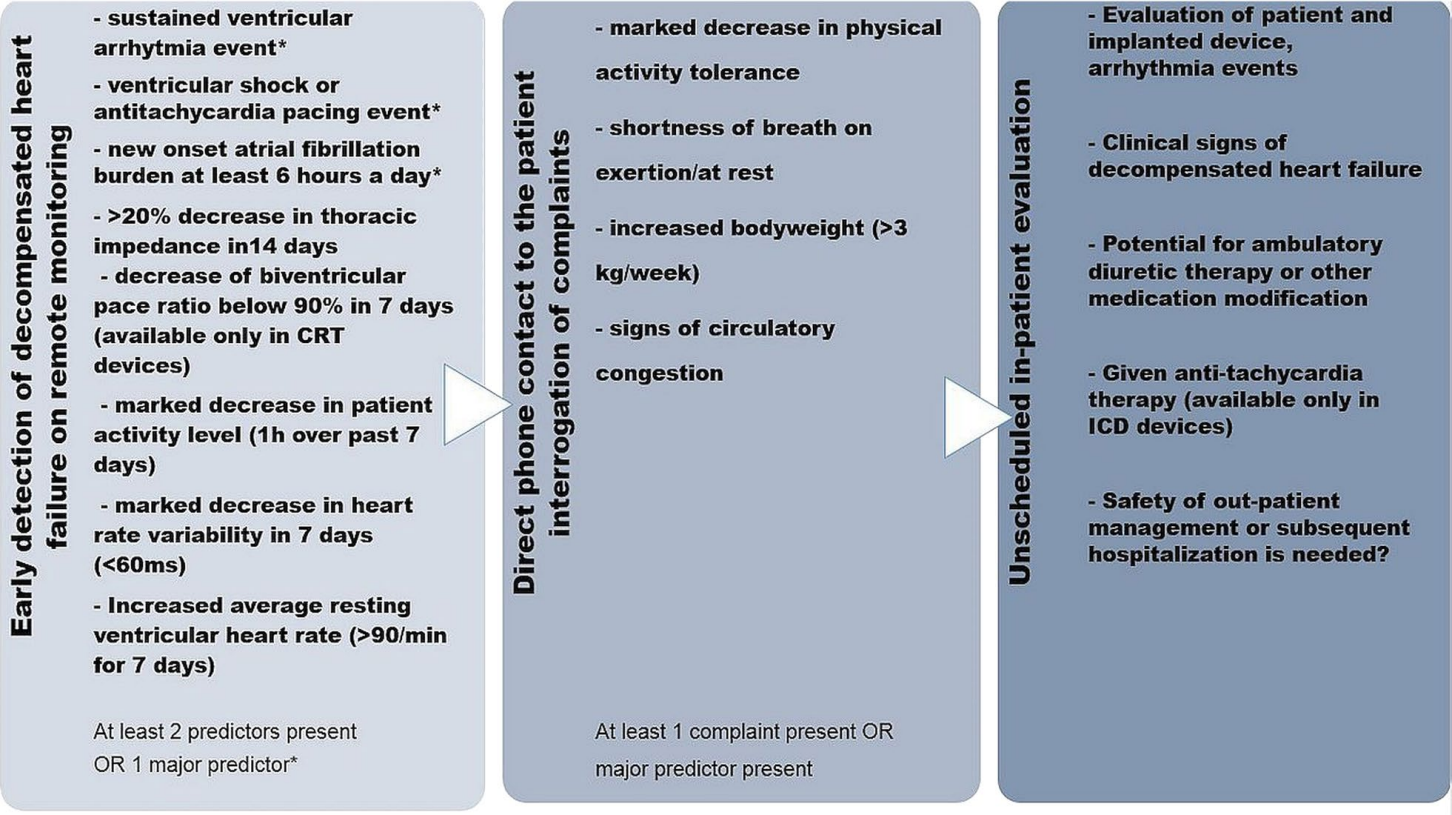
Preemptive detection of worsening heart failure related adverse events with the help of a predefined alert based workflow. Major criteria in the alert based detection algorithm were sustained ventricular arrhythmia or ventricular shock event, anti-tachycardia pacing event or new onset atrial fibrillation burden exceeding 6 h a day. At least two minor detection criteria positivity resulted in a consecutive telephone contact to the patient. Unscheduled in-office patient evaluations were arranged at major criteria positivity and/or at least 2 minor criteria positivity and presence of patient complaint

*Home Monitoring™* and *Care Link™* remote monitoring systems transmit automatically prespecified data to a manufacturer-specific server. The hospitals staff (cardiologists, electrophysiologists, trained nurse) responsible for the patient’s care can assess information on a secure website, where the patients are automatically classified and may flagged for clinical attention. Additionally, physicians are notified on prespecified alerts. Figure 1 shows specified remote monitoring detection algorithm and workflow in our institute for RPM capable CIED patients. Detection alerts were inspired from previous PARTNERS HF study [6] and optimalized on previous findings and clinical experience [7, 17].

Early detection of worsening heart failure was implemented by specific heart failure detection algorithms of Biotronik and Medtronic devices, general considerations are shown on Fig. 1. Monitoring data trends and alerting events were revised in weekly frequency.

Recent decrease in thoracic impendance value or increase (60 Ohms <) in Optivol™ value, decrease in heart rate variability, patient activity level, increasing resting heart rate, sustainded ventricular and supraventricular arrhthymia events and decrease in biventricular pacing ratio in resynchronization devices served as additive information about heart failure status in RMG.

In case of at least one major (sustained ventricular arrhtyhmia, anti-tachycardia therapy, new onset-, high ventricular rate atrial fibrillation events > 6 h a day) or in case of at least two minor alert positivity any potential patient symptoms were directly interrogated by a telephone contact and further heart failure related complaints were assessed.

Among CFG patients none of CIED or non-CIED remote monitoring activities were implemented, however patients were contacted on telephone by the device ambulance physician to assess potential complaints on abandoned IPE appointments. Unscheduled in-office visit events were exceptionally arranged on physician or general practinioner referral. In this cases IPEs were strongly complaint and symptom-based in this patient group.

### Study endpoints

The objective in this study was to compare adverse event rates in primary composite end-point of sustained arrhythmia- device- and worsening heart failure related adverse events in the patient groups during 12 months of COVID-19 pandemic.

The secondary end-point was to assess rates for worsening heart failure related hospitalizations in the two patient groups.

### Adverse event definitions

Sustained ventricular and/or supraventricular arrhythmia (ventricular tachycardia, fibrillation, atrial fibrillation) events requiring further treatment or appropriate/inappropriate ventricular shock events, new-onset- IPE or hospitalization necessitating arrhythmias were collected as arryhtyhmic adverse events in both patient groups.

Multiple alert signs of potential device dysfunction (abrupty elevated pacing threshold, out of range pacing- or shock impedance value, low battery status, over/undersensing etc.) were monitored continuously and marked as device related adverse events.

Even nowadays the definition of worsening heart failure (WHF) event is not universally accepted [18] and this definition bias can lead consequentially to inproper event assesment, so we defined worsening heart failure event as at least one grade deterioration in New York Heart Association heart failure functional class (NYHA class) from baseline and further need of parenteral diuretic- and other medical therapy intensification because of heart failure symptoms.

### Statistical analysis

The sample size calculation was based on a hypothesis, with a 25% margin for the occurrence of heart failure, arrhythmia and device related adverse events at 12-month follow-up assumed. Pre-set values were 5% for the significance level and 80% for the power. A required sample size of (54 + 54) 108 patients with complete datasets was calculated in an observational study design. After considering rate of incomplete data sets (predicted at approximately 10%), a total of ~ 130 patients were planned for recruitment.

All follow-up variables were divided to categorical or continuous variables. Data are presented as mean ± standard deviation for normally distributed continuous variables, median (25th and 75th percentiles) for non-normally distributed variables, or percentages for binary variables. Missing data were not replaced; all available data were used for sample distribution evaluation. Normality was checked with the Shapiro–Wilk test. For normal distributed data Student t test was used. Mann–Whitney test was used for inter-individual comparisons of continuous variables, when normality was rejected. Categorical variables were compared with the Chi-square or Fishers exact test. For primary endpoint outcome an adverse event free survival analysis was applied in Kaplan–Meier’s survival curve estimation with log-rank test. Spearman’s Rho correlation test was performed and binary logistic regressional analysis were performed to confirm ststistically significant correlations.

Statistical analysis was performed using IBM SPSS statistical software version 25.0. (Armonk, NY, IBM Corp.). The level of significancy was defined as *p* < 0.05.

## Results

### Patient populations

61 patients in the remote monitoring group (RMG) and 71 patients in the conventionally followed group (CFG) were involved in this observational study. Baseline patient characteristics of the two patient-groups are shown or Table 1.

**Table 1:** Baseline patient parameters

	Remote monitoring group (RMG), n = 61	Conventionally followed group (CFG), n = 71	*p* value
Age (years), median (IQR)	72.0 (61.5–77.5)	71.0 (59.0–77.0)	0.549
Sex (male/female)	46/15	54/17	0.931
Single chamber ICD, n (%)	27 (44.3)	29 (40.8)	0.291
Dual chamber ICD, n (%)	7 (11.5)	17 (23.9)	
CRT-defibrillator, n (%)	18 (29.5)	22 (30.1)	0.854
CRT-pacemaker, n (%)	9 (14.6)	3 (4.2)	**0.037**
ICD for secondary prevention of SCD, n (%)	16 (26.2)	17 (23.9)	0.763
Implantation time before study inclusion months (mean ± SD)	26.5 ± 10.3	28.3 ± 12.4	0.831
*Comorbidities:*			
Hypertension, n (%)	55 (90.2)	56 (78.9)	0.078
Diabetes, n (%)	30 (49.2)	34 (47.8)	0.235
Dyslipidaemia, n (%)	33 (54.1)	36 (50.7)	0.297
Atrial fibrillation, n (%)	24 (39.3)	22 (32.4)	0.410
NYHA class, n (%)	II: 16 (26.2)	II: 48 (66.2)	** < 0.001**
	III: 45 (73.8)	III: 23 (33.8)	
Chronic kidney disease, n (%)	15 (24.6)	12 (16.9)	0.277
Chronic lung disease, n (%)	12 (19.7)	15 (21.1)	0.837
Ischemic heart disease, n (%)	39 (63.9)	43 (60.6)	0.692
Previous myocardial infarction, n (%)	33 (54.1)	18 (25.4)	**0.001**
Previous open heart surgery	18 (31.6)	21 (32.4)	0.922
*LV systolic function/diameter:*			
LVEF, median (IQR)	35.0 (30.0–48.0)	38.0 (31.0–45.0)	0.073
LV EDD, median (IQR)	62.0 (54.00–65.0)	59.0 (56.0–68.5)	0.980
LV ESD, median (IQR)	45.0 (43.0–50.0)	45.5 (41.0–50.5)	0.852
*Medications:*			
ACEi/ARB (%)	95.1	80.28	**0.048**
ARNI (%)	4.9	12.7	**0.036**
BB (%)	95.1	100.0	0.065
MRA (%)	59.0	59.1	0.32
Amiodarone (%)	34.4	36.8	0.201
Antiplatelet agent (%)	55.7	38.2	**0.047**
OAC (%)	44.3	47.1	0.751
Statin (%)	55.1	43.6	**0.041**

*ICD* Implantable cardioverter defibrillator; *CRT* Cardiac resynchronization therapy; *SCD* Sudden cardiac death; *NYHA* New York Heart Association; *LV* Left ventricular; *LVEF* Left ventricular ejection fraction; *EDD* End-diastolic diameter; *ESD* End-systolic diameter; *ACEi* Angiotensin converting enzyme-inhibitor; *ARB* Angiotensin receptor blocker; *ARNI* Angiotensin receptor blocker/nephrilysin inhibitor; *BB* Beta receptor blocker; *MRA* Mineralocorticoid receptor antagonist; *OAC* Oral anticoagulant. The level of significance was defined as *p* < 0.05 (bold)

### Burden of in-office patient evaluations during COVID-19 pandemic

During the first 6 months of COVID-19 pandemic (15.03.2020 – 15.09.2020) the number of total in-office patient evaluations (IPE) in in our cardiac device ambulance decreased to 72% of the year before (1590 IPE to 1224 IPE; *p* = 0.032) and the total IPE number remained significantly decreased in the second 6 months (16.09.2020–15.03.2021) aswell with 88% of the investigations and device interrogations the year before (1581 IPE to 1392 IPE).

There were 37 IPE; 0.606 IPE/patient in RMG and 42 IPE; 0.591 IPE/patient in the CFG during the 12 months of follow-up period as shown on Table 2. No differences were observed in abandoned scheduled IPEs (0.6557 IPE/patient vs. 0.6197 IPE/patient; *p* = 0.633) or urgent, unscheduled IPE events (0.6065 IPE/patient vs. 0.5915 IPE/patient; *p* = 0.855).

**Table 2:** Event rates in patient groups at 6 and 12 months of follow up

	COVID-19 pandemic first 6 months			COVID-19 pandemic at 12 months		
	RMG	CFG	*p*	RMG	CFG	*p*
Arrhythmia and device related event (event/patient)	0.131	0.14	0.132	0.146	0.169	0.699
Arrhythmia and device related hospitalization (event/patient)	0.049	0.07	0.629	0.131	0.098	0.547
Worsening of heart failure event (event/patient)	0.231	0.145	0.069	0.328	0.267	0.151
Worsening of heart failure related hospitalization (event/patient)	0.016	0.169	**0.012**	0.115	0.225	0.096
Total in-office patient evaluations (event/patient)	0.262	0.253	0.98	0.606	0.591	0.959

*COVID-19* Corona virus disease 2019, *RMG* Remote monitoring group, *CFG* Conventionally followed group. The level of significance was defined as *p* < 0.05 (bold)

### Adverse event rates and hospitalization for heart failure

**Fig. 2:**
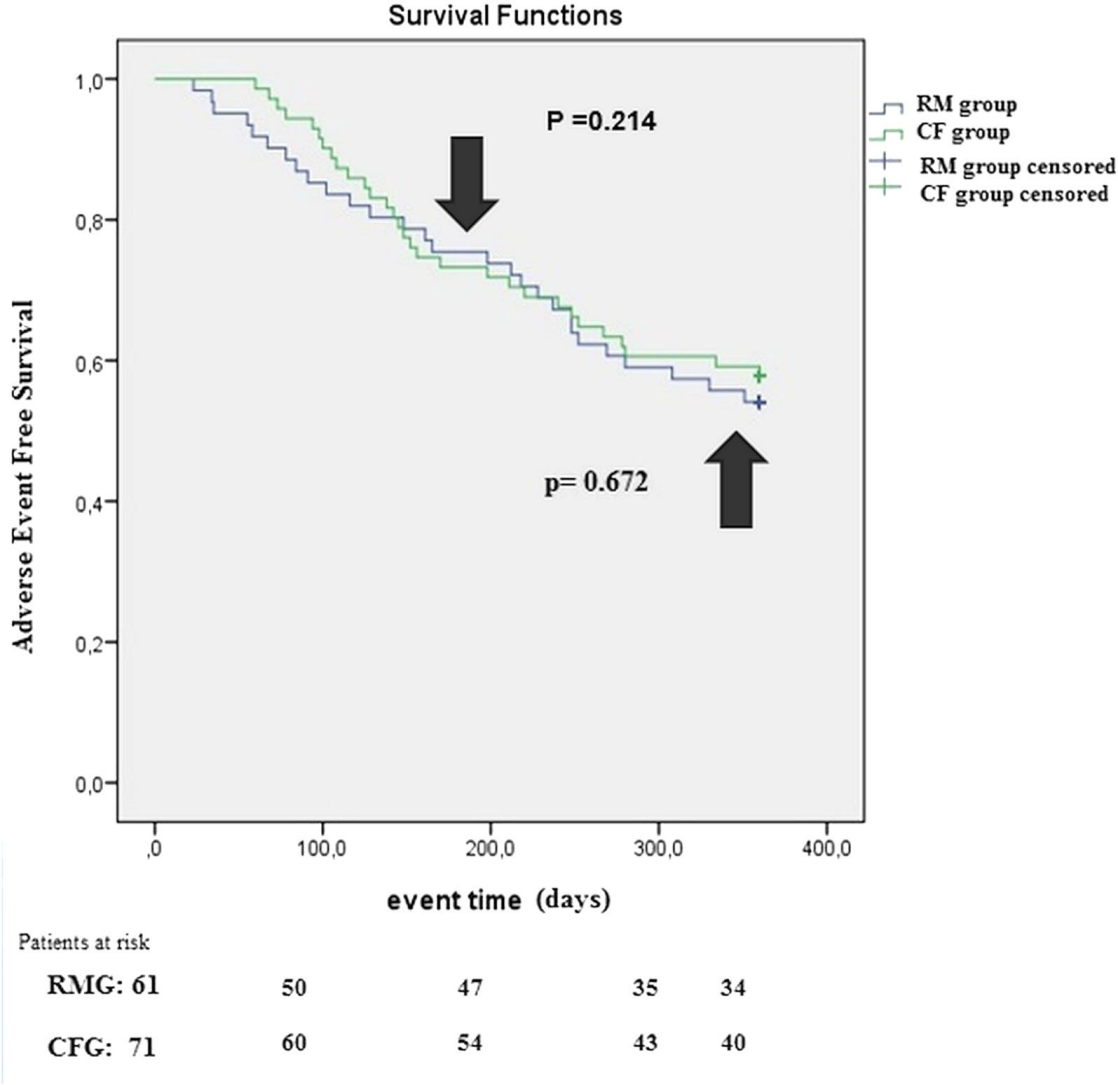
Kaplan-Meiers curve: Adverse event-free survival. The composite end-point of device-, arrhythmia and worseing heart failure related adverse event-free survival is statistically non-differeing in the two observed patient groups neither at 180 days (log rank *p* = 0.214) nor at 360 days (log rank *p* = 0.672) of follow-up during the COVID-19 pandemic

No statistically significant differences were seen neither at first 6 months (*p* = 0.214) nor 12 months (*p* = 0.672) in the primary composite end-point of device related-, arrhythmia- or worsening heart failure related adverse events between the two observed patient groups. Kaplan–Meier curve represents adverse event-free survival in the investigated patient groups during the observational period as shown on Fig. 2.

Worsening heart failure events in the RMG showed a statistically not significant but increased tendency (0.231 event/patient vs. 0.145 event/patient; *p* = 0.069) in the first 6 months of COVID-19 pandemic. In-spite of the upper tendency, the hospitalization numbers for worsening heart failure in the first 6 months of the pandemic were significantly lower in the RMG (0.016 event/patient vs. 0.169 event; *p* = 0.012) than in CFG (Table 2).

**Fig. 3:**
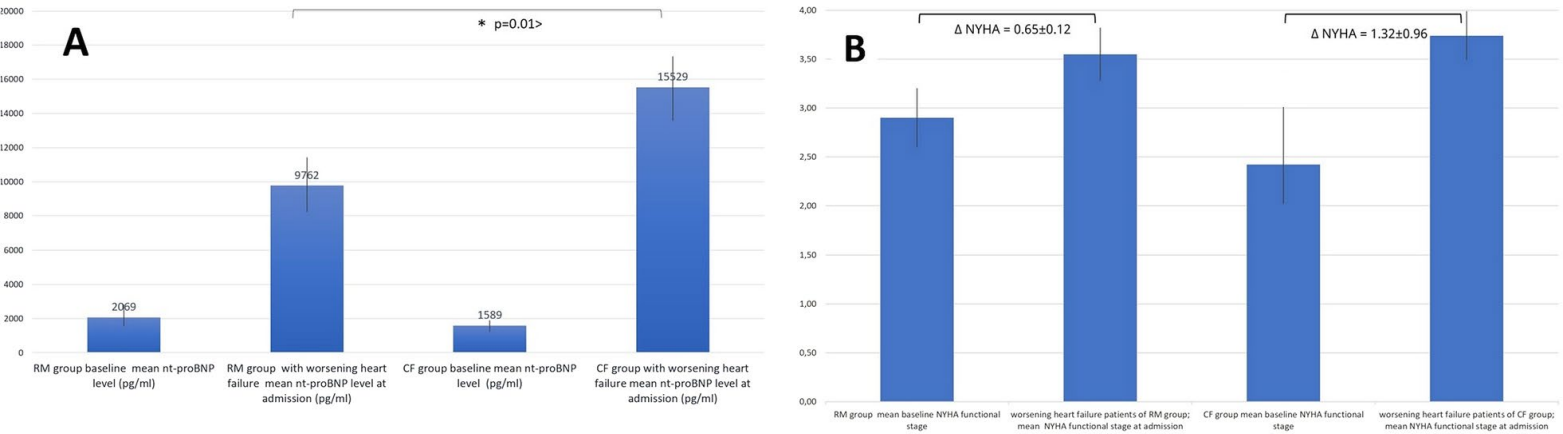
Comparison of NT-proBNP levels (**A**) and change in NYHA functional class (**B**) at baseline and hospital admissions for worsening heart failure in the remote monitoring (RMG) and conventionally followed (CFG) patient groups. Patients in the conventionally followed group (CFG) had a significantly increased N terminal-proBNP (brain natriuretic peptide) levels at worsening heart failure event related hospital admissions (15,529 ± 362 pg/ml in CFG vs. 9762 ± 168 pg/ml in the RMG; *p* = 0.01 >) and more pronounced deterioration from baseline NYHA functional class than patients in remote monitoring group (RMG) (mean ∆NYHA in CFG: 1.32 vs. mean ∆NYHA in RMG:0.65; *p* = 0.026)

Notably; patients with worsening heart failure event in CFG requiring in-office patient evaluation and/or hospitalization had significantly increased N terminal-proBNP (brain natriuretic peptide) levels (15,529 ± 362 pg/ml in CFG vs. 9762 ± 368 pg/ml in the RMG; *p* = 0.01 >) and more deterioration from baseline NYHA functional class than patients in RMG (mean ∆NYHA in RMG: 0.65 ± 0.12 vs. mean ∆NYHA in CFG: 1.32 ± 0.96; *p* = 0.026) as shown on Fig. 3A, B. Post-hoc power analysis calculation for overall hospitalization outcome showed 98.9% and for worsening heart failure associated hospitalization 86% statistical power with 0.05 value of alpha.

Arrhythmia events in the RMG were 2 cases of cumulated ventricular fibrillation/sustained ventricular tachycardia episodes and 2 cases of inappropriate ventricular shock due to high ventricular rate atrial fibrillation.

In the CFG 3 cases of cumulated sustained ventricular arrhythmia with or without adequate device therapy, 4 cases of atrial fibrillation with rapid venticular heart rate and 2 cases of inappropriate ventircular shocks were observed in the CFG. In one case of inappropriate ventircular shock atrial fibrillation with fast ventricular rate occured and in the other case shock-electrode impairment and noise oversensing was the underlying cause.

### Correlational analysis

Spearman’s rho correlational analysis and binary logistic regressional analysis showed statistical correlation for wosening heart failure events in patients with permanent atrial fibrillation (*p* = 0.025), higher baseline NYHA functional class (*p* = 0.037), decreased left ventricular ejection fraction (*p* < 0.001), increased left ventricular end-diastolic (*p* < 0.01) and end-systolic (*p* < 0.01) diameters.

Patients with permanent atrial fibrillation (*p* = 0.018), increased left ventricular end-diastolic (*p* < 0.01) and end-systolic diamters (*p* < 0.01) and decreased left ventricular ejection fraction (*p* < 0.01) had independently higher risk for hospitalization for worsening heart failure.

It has to be emphasized, that patients with specified remote monitoring alert-based follow-up scheme had independently lower risk for heart failure hospitalization (*p* = 0.045) in the observed 12 months of pandemic period.

## Discussion

Significant number of IPEs in device clinics were abandoned worldwide during COVID-19 pandemic, thus patients with automatic transmission based remote monitoring survelliance had potential advantage in the timely detection of clinically relevant adverse events with the help of previously developed alert-based follow-up models. Few of these remote follow-up modalities offer preemptive detection of worsening heart failure status of the patient [6, 7, 17]. There is a persisting need for a sophisticated and universally accepted automatic data transmission based monitoring system for predicting heart failure deterioration in CIED patients. Recently D’Onofrio et al. introduced a validated multiparameter monitoring based prediction algorithm for heart failure hospitalizations in SELENE HF (Selection of potential predictors of worsening heart failure) trial [19]. A basline risk-stratifier Seattle HF Model was combined with temporal trend of various physiological (diurinal- and nocturnal heart rate, heart rate variability, physical activity) arrhythmia (ventricular extrasystoles, atrial fibrillation burden) and thoracic impedance parameters. Reaching the nominal index threshold of the algorithm, patients had substantially increased risk for heart failure hospitalization. The algorithm was showed to have an 65.5% sensitivity for an upcoming heart failure event with acceptable false/unexplained alert rate of 0.69 alert/patient/year.

The primary end-pont of our observational study was to asses the composite end-point of arrhythmia, device and worsening heart failure realted adverse events in the two patient cohorts. These event rates were higher in our patient groups compared to an observational study which combined anti-bradycardia, ICD and CRT implanted patients during the SARS Cov-2 pandemic related lockdown in Italy. [20] Patients involved in our study had more advanced heart failure, this may explain relative higher observed adverse event rates. In addition the two involved patient populations in our study were non-homologous in terms of baseline patient comorbiditites, heart failure conditions and medications. Patients in RMG had worse baseline NYHA heart failure functional class and fewer patients were on ARNI (angiotensin receptor blocker/nephrilisin inhibitor) therapy. RMG patients had tendeciously higher risk for worsening heart failure event in the first 6 months of COVID-19 pandemic, where institutional restrictions were the most pronounced with a significant 28% decrease in the device interrogations and heart failure IPE numbers. Although tendenciously higher heart failure deteriorations were observed, these patients had only modest increase in NT-proBNP levels and suffered less deterioration in NYHA functional class compared to CFG patients. These results let us conclude that RMG patients who had worsening of heart failure had accelerated institutional detection and admission time. Preemptive detection and early pharmacological/non-pharmacological interventions at IPEs efficiently prevented further progression in heart failure status and hence reduced hospitalizations driven by decompensated heart failure. At 12 months follow-up time the upper seemed benefits in the RMG diminished and it might be explained by the baseline relevant differences between the two patient populations.

## Conclusions

We can conclude that alert based remote monitoring of CIED patients with advanced heart failure in our observational study enabled preemptive detection and fast clinical intervention at impeding cardiac decompensation events. Remote monitoring seems to play promising role in reducing the burden of heart failure hospitalizations even in pandemic circumstances. Further observational trials with larger patient populations are needed to confirm our findings.

## Data Availability

The datasets generated and/or analysed during the current study are not publicly available due patient privacy issues but are available from the corresponding author on reasonable request.

## Abbreviations

ARNI: Angiotensin receptor blocker/nephrilisin inhibitor
COVID-19: Coronavirus disease 2019
CFG: Conventionally followed group
CRT-P/D: Cardiac resynchroinzation therapy pacemaker/defibrillator
CIED: Cardiac implantable electronic device
ICD: Implantable cardioverter defibrillator
IPE: In-office patient evaluation
NT pro-BNP: N terminal pro-brain natriuretic peptide
NYHA: New York heart association functional class
RMG: Remote monitoring group
RPM: Remote patient management
Sars Cov-2: Severe acute respiratory syndrome coronavirus type 2
WHF: Worsening of heart failure
